# Unveiling the Role of Electronic, Vibrational, and Optical Features of the 1T′ WSe_2_ Monolayer

**DOI:** 10.1021/acsomega.4c07519

**Published:** 2024-09-28

**Authors:** Rafael Salles Barbosa, Celso Alves do Nascimento Júnior, Alexandre Silva Santos, Maurício Jeomar Piotrowski, Celso Ricardo Caldeira Rêgo, Diego Guedes-Sobrinho, David Lima Azevedo, Alexandre Cavalheiro Dias

**Affiliations:** † Institute of Physics, University of Brasília, Brasília 70919-970, Federal District, Brazil; ‡ Department of Physics, Federal University of Pelotas, P.O. Box 354, Pelotas 96010-900, Rio Grande do Sul, Brazil; § Karlsruhe Institute of Technology (KIT), Institute of Nanotechnology, Eggenstein-Leopoldshafen 76344, Germany; ∥ Chemistry Department, Federal University of Paraná, Curitiba CEP 81531-980, Brazil; ⊥ Institute of Physics and International Center of Physics, University of Brasília, Brasília 70919-970, Federal District, Brazil

## Abstract

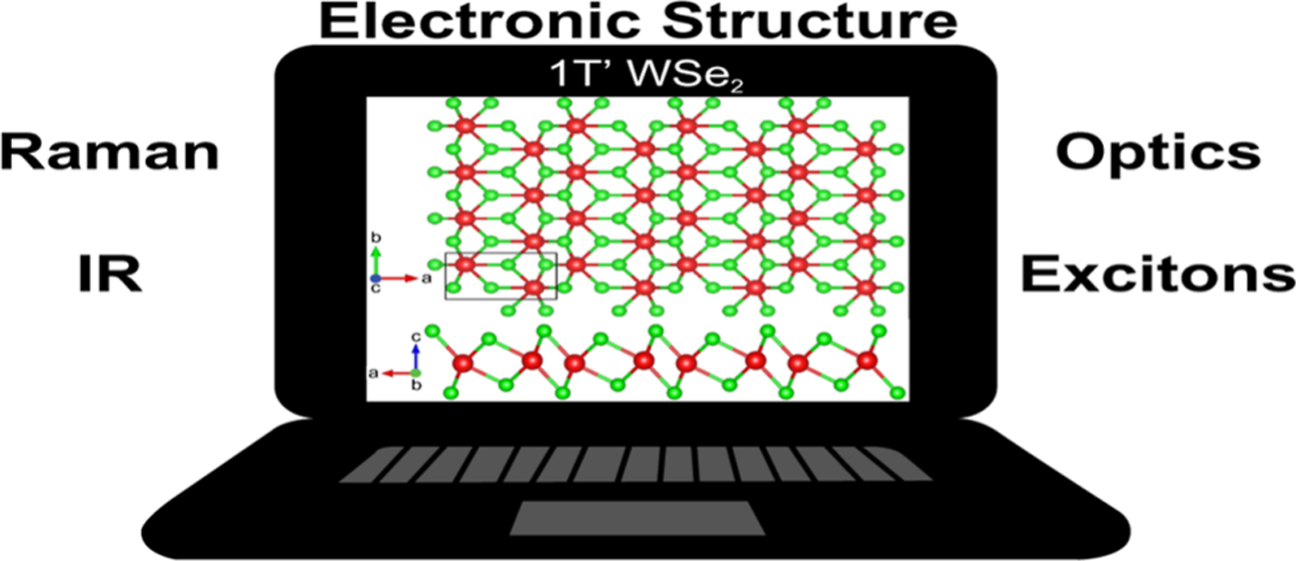

Understanding the optoelectronic profile and chemical stability of transition-metal dichalcogenides (TMDs) is crucial for advancing two-dimensional (2D) material applications, particularly in electronics, optoelectronics, and energy devices. Here, we investigate the structural, electronic, optical, and excitonic properties of the 1T′ WSe_2_ monolayer. Phonon dispersion analysis confirmed the thermodynamic stability of this system. The 1T′ WSe_2_ monolayer exhibits a small electronic band gap of 0.17 eV, and its linear optical response suggests the potential use as a polarizing filter due to its strong reflectivity at ŷ light polarization. Unlike the 1T′ MoS_2_ system, 1T′ WSe_2_ does not show an excitonic insulator phase. Instead, its exciton binding energy of 150 meV is consistent with values expected for 2D materials. This distinction underscores the unique electronic and optical properties of 1T′ WSe_2_, positioning it as a promising candidate for advanced technological applications such as flexible electronics, photodetectors, and quantum computing. By exploring these properties, we can unlock the full potential of TMDs in creating innovative high-performance devices.

## 1 Introduction

The discovery of graphene in the early 21st century^1,2^ opened new frontiers in materials research by focusing on 2D materials and their unique properties arising from quantum confinement in their nonperiodic direction.^3^ This new research area has unveiled a range of extraordinary properties^4,5^ and functionalities^6−8^ due to the atomic thickness of 2D materials, which allows for horizontal patterning through chemical and mechanical techniques.^9^ This also enables the combination of monolayers into van der Waals (vdW) heterojunctions, leading to a wide variety of properties.^10−12^ However, the dielectric profile of 2D materials and quantum confinement effects make excitonic properties, based on electron–hole (e–h) pairwise interactions, crucial for optical characterization, requiring sophisticated experimental and theoretical approaches.

**Table 1:** Frequencies and Symmetries of Raman and IR Vibrational Active Modes in 1T′ WSe_2_ Monolayer^27,50,51^^a^

frequency (cm^–1^)	Raman	IR
72.17	^1^A_g_	
98.47	^1^B_g_	
137.00	^2^B_g_	
144.03		^1^B_u_
145.70	^2^A_g_	
150.15	^3^B_g_	
165.31		^1^A_u_
169.22	^3^A_g_	
173.32	^4^B_g_	
177.25		^2^A_u_
204.10		^3^B_u_
207.53	^4^A_g_	
231.51	^5^B_g_	
253.86	^5^A_g_	
294.66	E′	E′

a The symmetry of Raman or IR inactive modes is left blank.

In this scenario, transition-metal dichalcogenides (TMDs) have been extensively investigated^7,8,13^ due to their atomic configurations, which feature a stable phase similar to graphene’s honeycomb structure, known as the 2H phase.^7^ The technological importance of TMDs is significant as their semiconducting properties make them ideal candidates for a wide range of applications, including flexible electronics, photodetectors, and energy storage devices.^14−16^ Their unique electronic, optical, and mechanical properties enable the development of high-performance transistors, sensors, and catalysts. The ability to form vdW heterojunctions further expands the potential for creating novel optoelectronic devices with tailored properties, such as light-emitting diodes and solar cells. By leveraging the atomic-scale thickness and the flexibility of TMDs, researchers can design next-generation technologies that are lightweight, highly efficient, and adaptable to various environments, thereby revolutionizing the field of materials science and engineering. Unlike graphene, which is a semimetal,^1,2^ TMDs exhibit semiconductor behavior. These materials consist of a transition metal (M) sandwiched between two chalcogen (X) atoms, with an MX_2_ stoichiometry, forming hexagonal layers. TMD monolayers present several polytypic structures, including 2H,^7^ 1T,^17^ and 1T’.^18,19^ It is known that for Mo- and W-based TMDs, the 1T phase is unstable in the free-standing condition,^17,20^ undergoing a stabilization process through a spontaneous lattice distortion (known as Peierls distortion) in the *x̂* direction, resulting in a 2 × 1 × 1 distorted supercell, also known as the 1T′ phase.^20^ This phase features 1D zigzag chains along the ŷ direction.^20^

The 1T′ phase has garnered significant attention due to its topological properties and potential applications in electronic and spintronic devices.^18,19^ The transition from the 1T phase to the 1T′ phase can be induced through methods such as chemical doping and pressure application for TMDs stable in the 1T phase,^21−23^ providing a versatile platform for exploring new physical phenomena and developing innovative technologies. Recent studies have proposed 1T′ Mo- and W-based TMD monolayers as candidates for the quantum spin Hall effect due to the overlap of metal-d conduction and chalcogenide-p valence bands.^18,24^ This band localization mechanism offers a straightforward way to control topological electronic properties using an external electric field, which is highly desirable for vdW devices.^18^ For instance, Varsano and co-workers have observed excitonic insulator behavior in 1T′ MoS_2_ monolayers.^24^

Even with the vast amount of literature references on TMDs, the 1T′ WSe_2_ monolayer count only with some few insights about its electronic properties.^18^ Thus, due to this lack, we comprehensively characterized its structure and stability and described its excitonic effects. Therefore, this work studies the structural, electronic, vibrational, optical, and excitonic properties of the 1T′ WSe_2_ monolayer. We started with phonon dispersion analysis to confirm the thermodynamic stability of this monolayer. From the phonon data, the thermodynamic properties were obtained, from which we can offer a set of physicochemical insights for the synthesis route based on a particular interval of temperature. The vibrational spectrum, which depends from the electronic structure and phonon dispersion, was used to validate our simulation with the current experimental data of 1T′ WSe_2_.^25−27^ Followed by an investigation of its electronic properties using Perdew–Burke–Ernzerhof (PBE) and Heyd–Scuseria–Ernzerhof (HSE06) functionals, including spin–orbit coupling (SOC) effects. Given the quantum confinement in the nonperiodic direction, excitonic effects are expected to be significant in these 2D systems.^7^ To better understand their role in optical characterization, we calculate the optical properties such as the absorption coefficient, refractive index, and reflectivity using the independent particle approximation (IPA) and by considering quasiparticle effects through the Bethe–Salpeter equation (BSE),^28^ complemented by Raman and infrared (IR) spectra. This comprehensive characterization underscores the importance of 1T′ WSe_2_ in advancing the field of 2D materials and its potential for technological applications.

## 2 Theoretical Methodology and Computational Details

We conducted the simulations using density functional theory (DFT),^29,30^ explicitly solving the Kohn–Sham (KS) equations with the Vienna ab initio simulation package (VASP)^31,32^ and employing the projector augmented-wave method.^33,34^ For the electronic and structural properties, we utilized the PBE functional,^35^ a semilocal exchange–correlation functional within the generalized gradient approximation.^36^ Recognizing the tendency of PBE to underestimate the electronic band gap due to self-interaction errors,^37−41^ we adopted the HSE06 hybrid functional^42,43^ for a more accurate band gap estimation. Considering the presence of tungsten (W) in the system, we included SOC effects. To ensure precision in the band structure calculations, we doubled the number of bands relative to the number of electrons in the system.^7^

We obtained the equilibrium lattice parameters through stress tensor optimization and atomic force minimization. For lattice optimization, we used a cutoff energy of 634.26 eV, while other properties used a cutoff energy of 356.78 eV. We converged the KS self-consistent cycle to a total energy criterion of 10^–6^ eV. We considered the equilibrium structures achieved when the atomic forces were below 0.010 eV Å^–1^. For Brillouin zone integration, we used a k-mesh of 7 × 12 × 1 for all electronic properties, except for the density of states, which required a higher k-mesh of 13 × 23 × 1. To prevent spurious interactions between the monolayer and its periodic images along the nonperiodic *z*-direction, we employed a vacuum thickness of 20 Å. We performed phonon dispersion calculations using VASP (PBE) and the Phonopy package,^44^ employing density functional perturbation theory with a 2 × 2 × 1 supercell and a 3 × 6 × 1 k-mesh.

We analyzed stability using off-resonance Raman activity, determined by the method developed by Porezag and co-workers,^45^ focusing on the phonon vibration modes at Γ. For these calculations, we used the computational approach implemented by Fonari and Stauffer.^46^ IR and Raman spectra are obtained using a Gaussian smearing of 1 cm^–1^. To investigate excitonic and optical properties, we applied the IPA and the BSE,^28^ with the latter incorporating excitonic quasi-particle effects. We utilized the WanTiBEXOS code^47^ for these calculations.

We described electronic single-particle levels using maximally localized Wannier functions tight binding (MLWF-TB), obtained from VASP HSE06 + SOC DFT simulations through the Wannier90 package,^48^ considering d-orbital projections from W and p-orbital projections from Se. We solved the BSE using the 2D Coulomb truncated potential (V2DT),^49^ considering the six lowest conduction bands and the ten highest valence bands, and employing a k-mesh of 20 × 36 × 1. We applied a smearing of 0.01 eV to the dielectric functions at both BSE and IPA levels to ensure accurate results.

## 3 Results and Discussion

### 3.1 Structural Stability and Thermodynamic Properties

**Figure 1:**
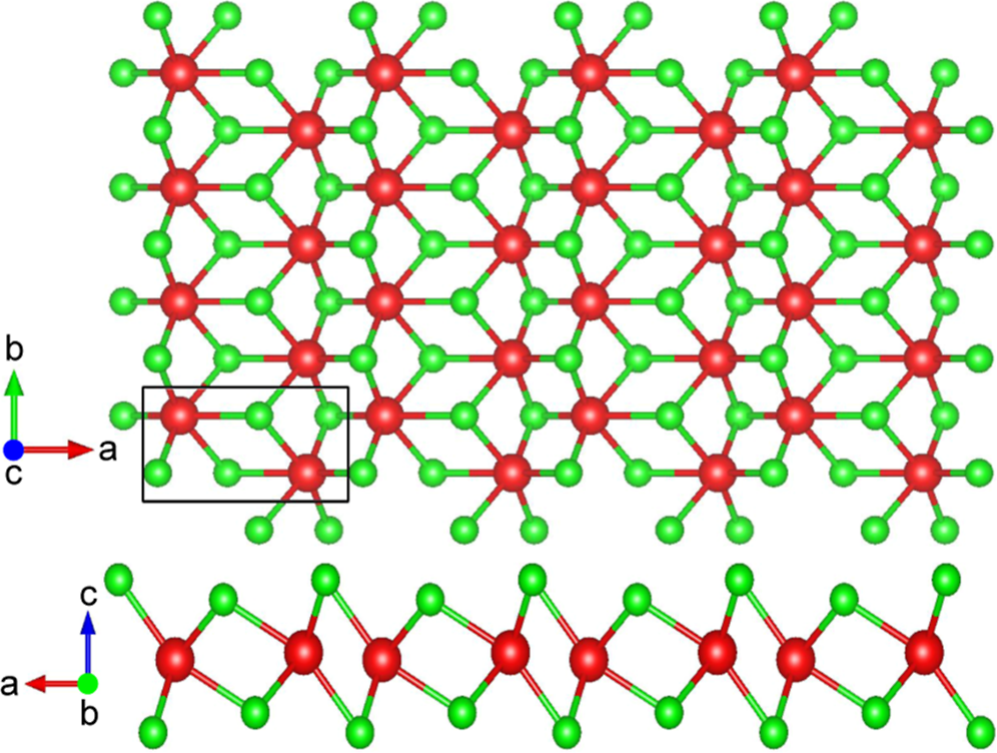
WSe_2_ 1T′ monolayer crystal structure, viewed from the top and the side. The W (red spheres) and Se (green spheres) atomic species are represented.

The WSe_2_ monolayer in the 1T′ phase has a rectangular unit cell consisting of 6 atoms: 2 W and 4 Se, as shown in Figure 1. It belongs to the monoclinic crystal system, specifically the space group *P*_21_/*m*, and has a *C*_2*h*_ point group.^27^ The results of the geometry optimization determined the lattice parameters to be *a*_0_ = 5.94 Å and *b*_0_ = 3.30 Å. The 1T′ designation indicates its unique crystallographic phase, characterized by distorted octahedral coordination similar to the 1T phase. This distortion arises from Peierls distortions, which result in varying W–Se bond lengths within the unit cell.

**Figure 2:**
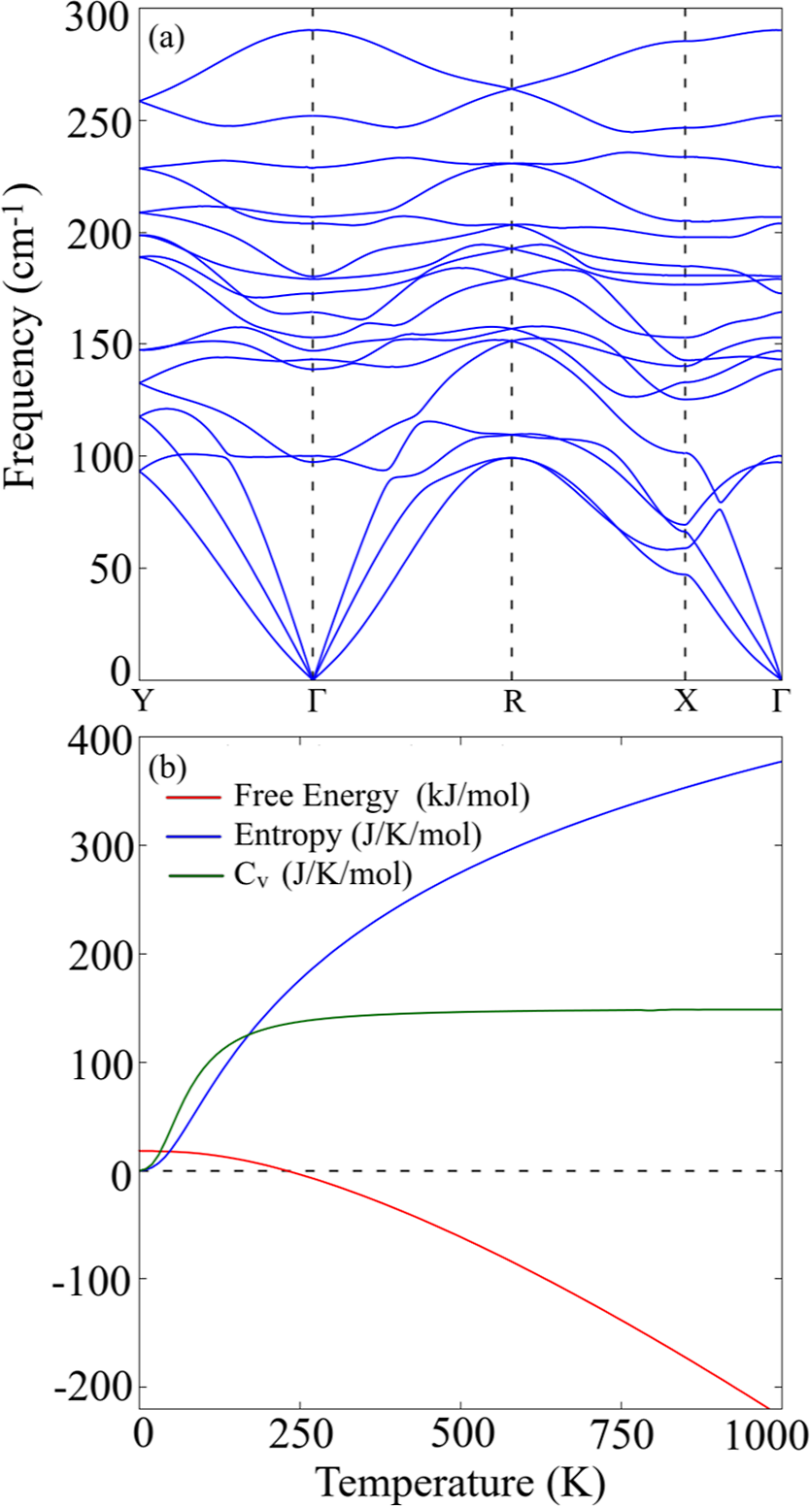
1T′ WSe_2_ monolayer phonons and thermodynamic properties: (a) phonon dispersion and (b) thermodynamic properties: Gibbs free energy (red curve), entropy (blue curve), and heat capacity at constant volume (green curve).

**Figure 3:**
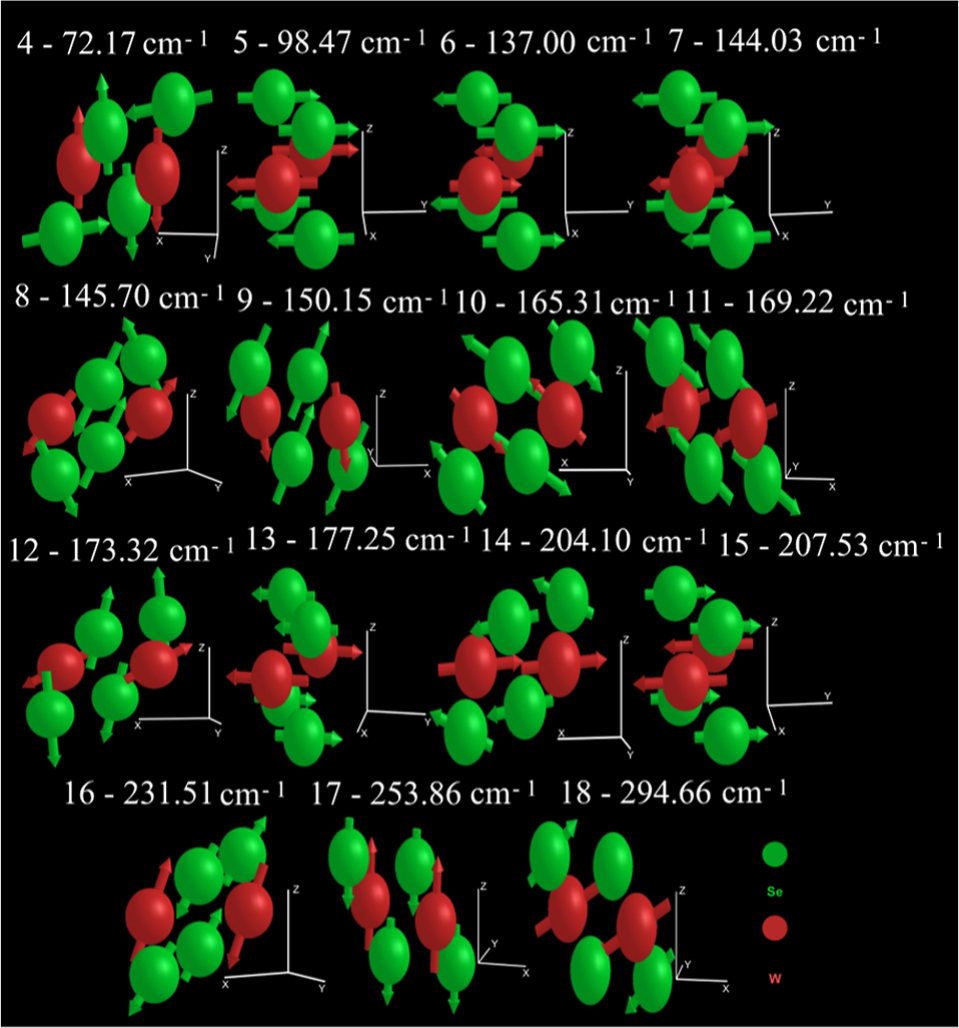
1T′ WSe_2_ monolayer phonon optical vibrational modes at the Γ point. The W (Se) atomic species are represented by the red (green) spheres.

As depicted in Figure 2a, the stability of the WSe_2_ monolayer is confirmed by the absence of imaginary frequencies in the phonon dispersion, ensuring its thermodynamic stability as a monolayer. The structure exhibits 18 vibrational modes: the first 3 are acoustic modes, where all atoms vibrate in unison, while the remaining 15 are optical modes, shown in Figure 3.

We examined thermodynamic stability using phonon dispersion within the harmonic approximation, as shown in Figure 2b. The plot includes the Gibbs free energy (red curves), entropy (blue curves), and heat capacity at constant volume (green curves). Entropy increases linearly up to 250 K and then becomes nonlinear. The heat capacity exhibits a *T*^3^ dependence at low temperatures (0 to 180 K) and approaches the Dulong–Petit limit around 500 K. The Gibbs free energy decreases with increasing temperature, becoming negative above 230 K, indicating favorable synthesis conditions at room temperature or higher for *T* > 230 K.

### 3.2 Raman and IR Spectra

**Figure 4:**
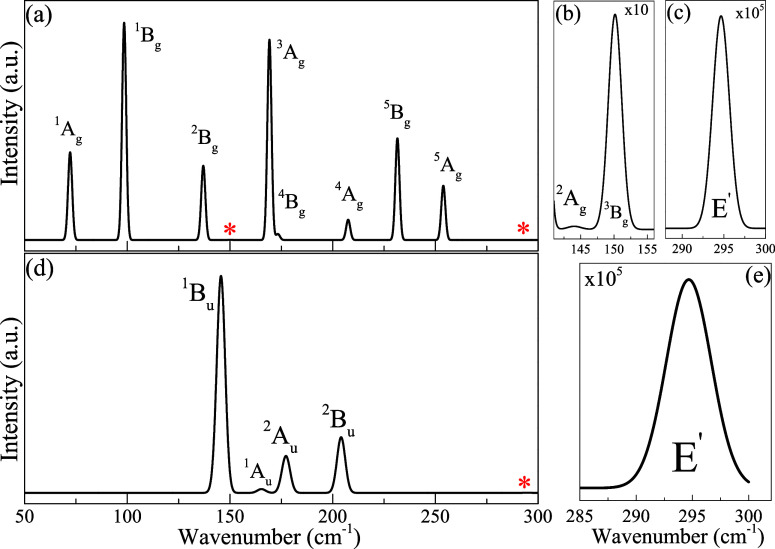
1T′ WSe_2_ monolayer: (a) Raman spectrum, zoomed-in regions from 140 to 160 cm^–1^ (b) and 280 to 300 cm^–1^ (c); (d) IR spectrum, zoomed-in region from 280 to 300 cm^–1^ (e).

To investigate the stability and dynamics of the 1T′ WSe_2_ monolayer, we calculated the Raman and IR spectra, presented in Figure 4a–e, respectively. Panels (b) and (c) focus on the 140 to 160 cm^–1^ and 280 to 300 cm^–1^ regions of the Raman spectrum, while panel (d) highlights the 280 to 300 cm^–1^ region of the IR spectrum. The monolayer exhibits 18 vibrational modes: 3 acoustic modes (inactive in Raman and IR due to their lack of significant polarizability) and 15 optical modes, as detailed in Figure 3. The irreducible representation of these vibrational modes is expressed as
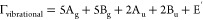
1where A_g_ and B_g_ modes are active in Raman spectroscopy, A_u_ and B_u_ modes are active in IR spectroscopy, and E′ modes are active in both.^25^ The group symmetry of each Raman and IR active modes are shown in Table 1.

In the Raman spectrum [Figure 4 panel (a)], peaks at 98.47, 137.00, 207.53, and 231.51 cm^–1^ correspond to antisymmetric in-plane vibrations, while other peaks represent antisymmetric out-of-plane vibrations. In the IR spectrum [Figure 4 panel (d)], peaks at 144.03, 165.31, 177.25, and 204.10 cm^–1^ indicate antisymmetric in-plane vibrations. The peak at 294.66 cm^–1^, associated with the E′ mode, is active in both Raman and IR spectra but is of low intensity, necessitating zoomed-in views for better visualization. Peaks marked with an asterisk in both spectra required zooming in due to their low intensity compared to that of other modes. All vibrational modes observed in the Raman and IR spectra are consistent with the phonon dispersion graph (Figures 2a and 3) and align with experimental results from Sokolikova et al.^27^ and Chen et al.^26^ and theoretical results from Cao et al.^52^

### 3.3 Electronic Properties

**Figure 5:**
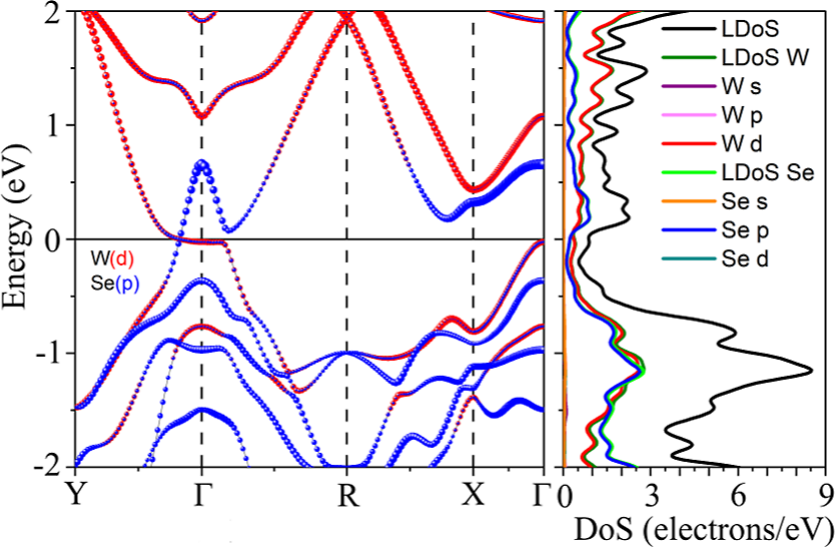
1T′ WSe_2_ monolayer orbital-projected band structure (left panel) and projected density of states (right panel) at the PBE level. The Fermi level is set at 0 eV.

From the orbital-projected band structure at the PBE level (Figure 5, left panel), the WSe_2_ monolayer exhibits metallic behavior, with the band gap closing between the *Y* and Γ high symmetry points. A small flat band region is observed between *Y* and *R*. The PBE orbital-projected density of states, shown around the Fermi level in the right panel of Figure 5, reveals that significant contributions come from W d-orbitals and Se p-orbitals, confirming that other orbitals have a minimal contribution around the Fermi level. This behavior is similar to that observed in 1T′ MoS_2_.^18^

**Figure 6:**
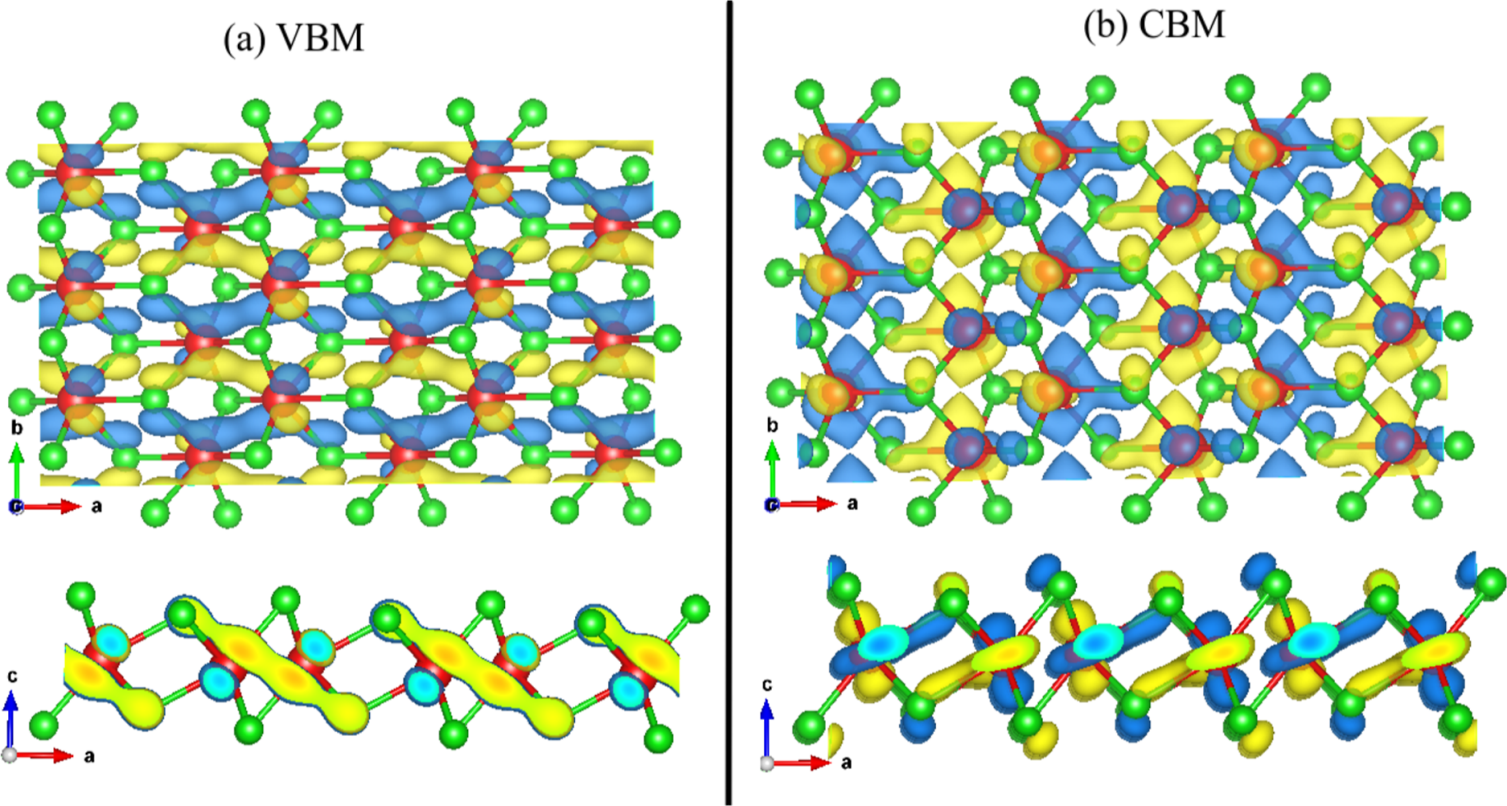
1T′ WSe_2_ monolayer top and side views of (a) VBM and (b) CBM wave functions.

The electronic wave functions of the valence band maximum (VBM) and conduction band minimum (CBM) are shown in Figure 6a,b, respectively. As seen, as depicted by the isosurface relative to the VBM at panel (a), one realizes an electronic localization predominance by contributing the W–Se diagonal bonds to the W plane. Since the VBM is confined throughout two units of WSe neighbors, it can justify the VBM flat band behavior around Γ. On the other hand, panel (b) depicts the CBM more delocalized, which explains the higher dispersion when compared with the VBM. At the same time, W sites present a predominance of the d orbitals, and at the same time, Se from both monolayer sides contributes with their p orbitals.

**Figure 7:**
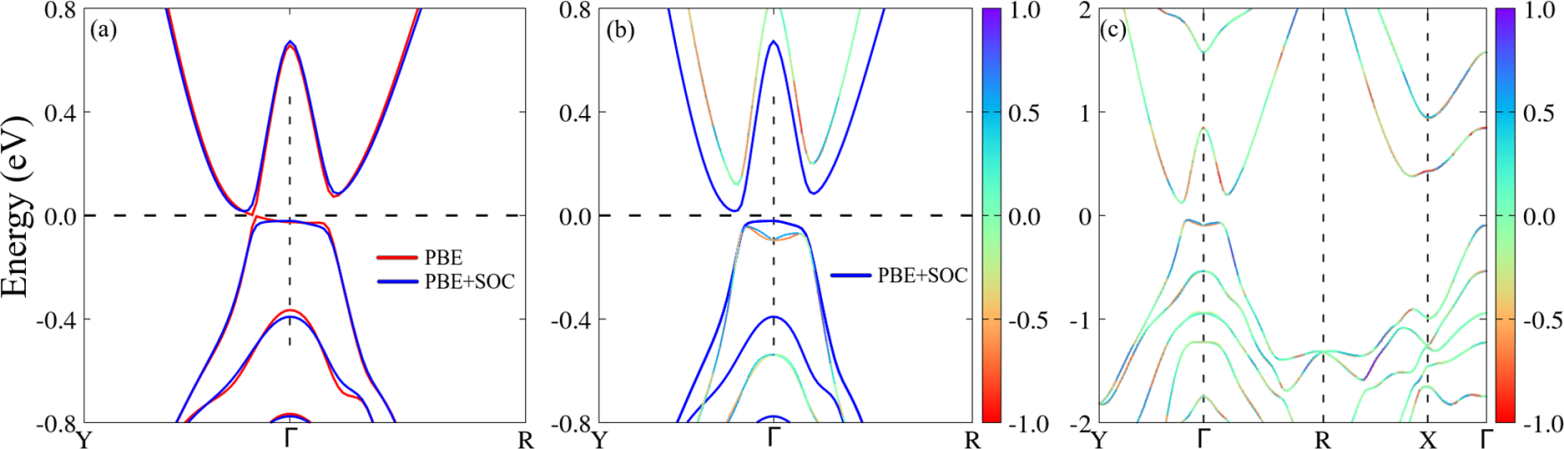
1T′ WSe_2_ monolayer band structure: (a) Zooming in on the band gap region comparing PBE (red curves) and PBE + SOC (blue curves) bands, (b) the same as (a) but comparing PBE + SOC (blue curves) with HSE06 + SOC (colored curves), and (c) HSE06+SOC in the Brillouin zone K-path. The color code in (b,c) represents the average value of the spin *s*_*z*_ component. The Fermi level is set at 0 eV.

In Figure 7, panel (a) compares the electronic band structures around the Fermi level using PBE (black curves) and PBE + SOC (red curves). Inclusion of SOC introduces a small indirect band gap of 0.11 eV, without a significant spin-splitting degeneracy breaking near the Fermi level. Panels (b), (c) compare the band structures obtained from PBE + SOC (black curves) and HSE06 + SOC (red curves). The hybrid exchange–correlation functional HSE06 enhances the electronic band gap, resulting in an indirect fundamental band gap of 0.17 eV and a direct band gap of 0.25 eV. A small spin-splitting in the valence band around the Γ point suggests a slight Rashba effect due to the Peierls distortion combined with higher SOC effects from the HSE06 functional. This observation aligns with previous studies^7^ on 2H polytype TMD monolayers.

### 3.4 Optical and Excitonic Properties

**Figure 8:**
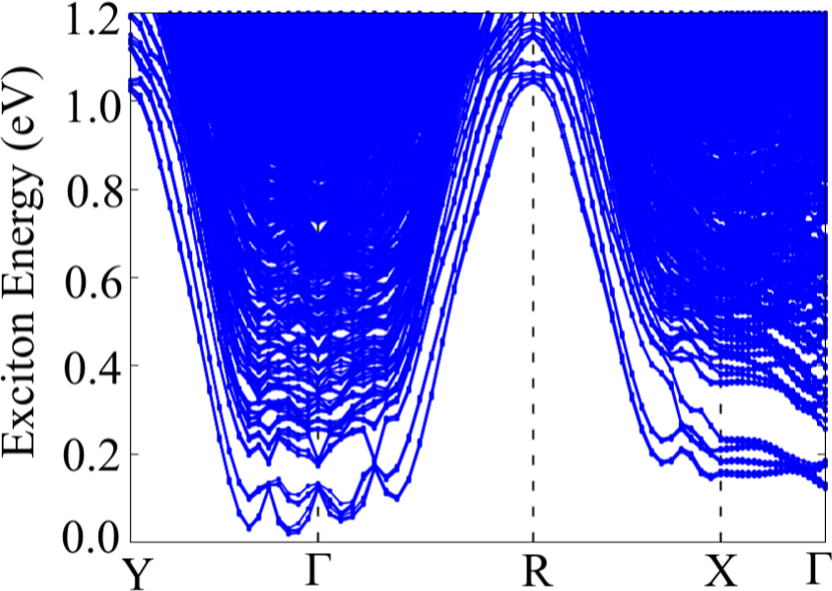
1T′ WSe_2_ monolayer exciton band structure obtained with MLWF-TB + BSE, HSE06 + SOC parametrization.

To further understand the excitonic properties of the 1T′ WSe_2_ monolayer, we calculated the excitonic band structure, shown in Figure 8. This structure includes excitonic states from direct (at Γ) and indirect (at other k-points) electron–hole pairs. Although it provides additional insights beyond the electronic band structure, it does not classify exciton bands as conduction or valence states. Excitonic levels result from the energy difference between the conduction and valence states minus the Coulomb interaction potential. The exciton ground state is indirect, with an energy of 0.02 eV, leading to an exciton binding energy of 150 meV, consistent with values expected for 2D materials.^7^ The direct excitonic ground state, corresponding to the optical band gap, is 0.12 eV. Unlike 1T′ MoS_2_, classified as an excitonic insulator due to its higher exciton binding energy than the electronic band gap,^24,53^ the 1T′ WSe_2_ monolayer is classified as a small band gap semiconductor.

**Figure 9:**
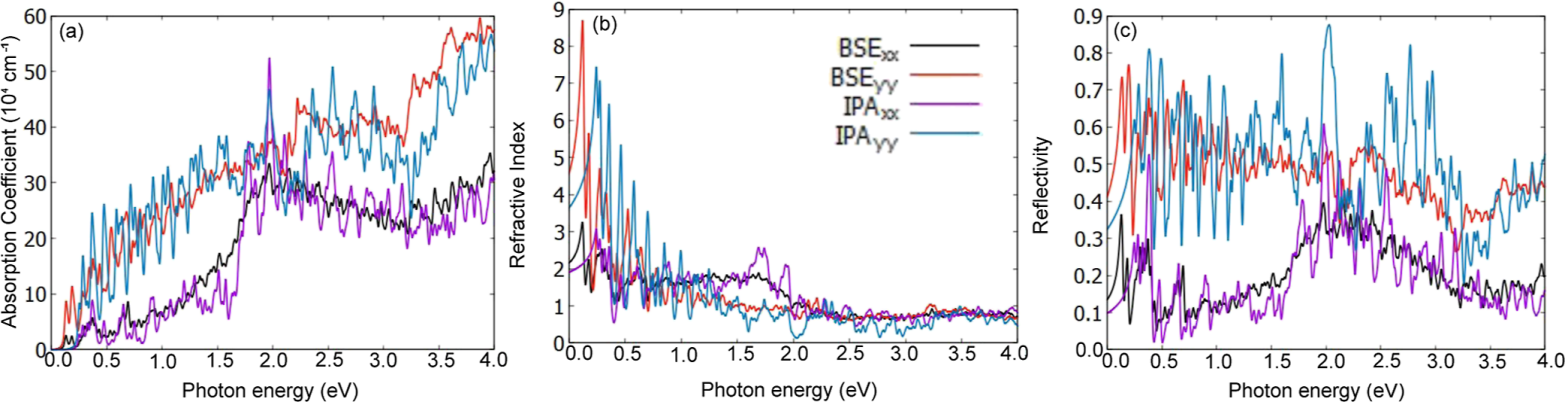
1T′ WSe_2_ monolayer optical properties: (a) absorption coefficient, (b) refractive index, and (c) reflectivity at BSE (red and black curves) and IPA (purple and blue curves) levels, considering linear *x̂* (black and purple curves) and *ŷ* (red and blue curves) light polarizations.

For optical characterization, we calculated the linear optical response (absorption coefficient) shown in Figure 9, with (black and red curves) and without (purple and blue curves) excitonic effects. Panel (a) displays the absorption spectrum, highlighting the optical anisotropy of 1T′ WSe_2_. The absorption coefficient varies significantly with the incident light polarization, being more intense for ŷ polarization. Despite an exciton binding energy of 150 meV, the primary change in the absorption coefficient due to quasi-particle effects is the appearance of two small peaks at 0.12 and 0.17 eV. At the IPA level, the optical band gap is 0.25 eV, with the rest of the absorption spectrum remaining relatively similar.

Panels (b,c) of Figure 9 present the refractive index and reflectivity, respectively. The refractive index for *ŷ* polarization is approximately three times higher than that for *x̂* polarization in the IR region. In the visible region, the values are closer to 1, indicating minimal deviation of incident light. The reflectivity shows significant anisotropy, ranging from 50% to 80% for *ŷ* polarization from 5% to 40% for *x̂* polarization across the solar emission spectrum (0.5 to 4.0 eV), suggesting potential application of this monolayer as a polarizing filter.

## 4 Conclusions

This study comprehensively characterized the structural, electronic, optical, and excitonic properties of the 1T′ WSe_2_ monolayer. Structurally, the system has a rectangular unit cell with lattice constants *a*_0_ = 5.94 Å and *b*_0_ = 3.30 Å. The phonon dispersion analysis confirmed the thermodynamic stability of the monolayer, suggesting the feasibility of experimental synthesis, particularly at temperatures above 230 K. The electronic property analysis revealed that the 1T′ WSe_2_ monolayer acts as a small band gap semiconductor with a band gap of 0.17 eV. We observed a small Rashba splitting around the Γ high-symmetry point, attributed to Peierls distortion combined with W SOC. The primary orbital contributions near the Fermi level are from W d orbitals and Se p orbitals.

The Raman and IR spectra provided valuable fingerprints for identifying vibrational modes associated with the material, facilitating comparison with experimental data and aiding future studies of interlayer interactions. In terms of excitonic and optical properties, unlike 1T′ MoS_2_, the 1T′ WSe_2_ monolayer is not an excitonic insulator. It is classified as a small band gap semiconductor with an optical band gap of 0.12 eV when excitonic effects are included and an exciton binding energy of 150 meV. The absorption coefficient reveals two small peaks at 0.12 and 0.17 eV due to quasi-particle effects. The system exhibits optical anisotropy, with more intense absorption for *ŷ* light polarization. This anisotropy is further evident in the refractive index and reflectivity. The pronounced reflectivity at *ŷ* polarization suggests potential applications of this monolayer as a polarizing filter.

These findings will inspire further research into the applications of 1T′ WSe_2_ in nanoelectronics and optoelectronics. At this stage, we aim to validate the present simulation protocol to explore a broader spectrum of TMDs. By leveraging the SimStack workflow framework,^54,55^ we can systematically create and execute comprehensive screening workflows based on these materials’ electronic, optical, and excitonic properties. This methodology will enable rapid prototyping and high-throughput testing of various TMDs, facilitating the identification of materials with optimal properties for advanced applications in flexible electronics, photodetectors, and energy storage devices. The integration of SimStack allows us to streamline the simulation processes, ensure reproducibility, and enhance the efficiency of the research efforts, thereby accelerating the development and deployment of innovative TMD-based technologies.^56−58^
